# Genome-wide whole blood microRNAome and transcriptome analyses reveal miRNA-mRNA regulated host response to foodborne pathogen *Salmonella* infection in swine

**DOI:** 10.1038/srep12620

**Published:** 2015-07-31

**Authors:** Hua Bao, Arun Kommadath, Guanxiang Liang, Xu Sun, Adriano S. Arantes, Christopher K. Tuggle, Shawn M.D. Bearson, Graham S. Plastow, Paul Stothard, Le Luo Guan

**Affiliations:** 1 Department of Agricultural, Food and Nutritional Science, University of Alberta, Edmonton, Alberta, T6G2P5 Canada; 2 Department of Animal Science, Iowa State University, Ames, IA, 50011 United States; 3 Food Safety & Enteric Pathogens Research Unit, USDA/ARS/National Animal Disease Center, Ames, Iowa, 50010 USA

## Abstract

To understand the role of miRNAs in regulating genes involved in host response to bacterial infection and shedding of foodborne pathogens, a systematic profiling of miRNAs and mRNAs from the whole blood of pigs upon *Salmonella* challenge was performed. A total of 62 miRNAs were differentially expressed post infection (false discovery rate <0.1). An integrative analysis of both the differentially expressed miRNAs and mRNAs using sequence-based miRNA target prediction and negative correlation of miRNA-mRNA profiles helped identify miRNA-mRNA networks that may potentially regulate host response to *Salmonella* infection. From these networks, miR-214 and miR-331-3p were identified as new candidates potentially associated with *Salmonella* infection. An miRNA seed sequence analysis suggested that these miRNAs regulate several critical immune-related genes including *SLC11A1*, *PIGE-108A11.3* and *VAV2*. We showed that challenged pigs had reduced miR-214 expression and increased miR-331-3p expression in the whole blood. Furthermore, the expression of the proposed targets of miR-214 (*SLC11A1* and *PIGE-108A11.3*) increased while that of the proposed target of miR-331-3p (*VAV2*) decreased following challenge (expression changes confirmed by *in vitro* assays). Based on these observations, we propose potential roles for miR-214 and miR-331-3p in regulation of immune responses to *Salmonella* infection.

*Salmonella enterica* serovar Typhimurium is a Gram-negative enteric pathogen which causes morbidity, mortality and economic loss worldwide^1^. *Salmonella* causes a wide range of systemic infections, including gastroenteritis, typhoid fever, bacteremia and endovascular infections^2^. Salmonelloses in humans, are usually acquired through the consumption of *Salmonella* contaminated pork products and through the direct contact with infected pigs^3^. In addition, salmonellosis in pigs contributes to significant economic losses to the pig industry^4^. To reduce the incidence and severity of salmonellosis and other infectious diseases, there is an urgent need to better understand the regulatory mechanisms underlying host immune response to *Salmonella* infection.

MicroRNAs (miRNAs) have been uncovered as key regulators of gene expression at the post-transcriptional level^5^,^6^,^7^. They control gene expression by regulating mRNA stability and translation in a wide range of biological processes including cell cycle, differentiation, apoptosis and disease pathogenesis^5^,^8^,^9^,^10^. There is increasing evidence that they have important roles in regulating innate immune response, the first line of defense to bacteria, viruses, and other pathogens^11^,^12^,^13^,^14^. A recent study using infected murine macrophages revealed that *Salmonella* strongly induces the NF-κB-induced miRNAs, miR-21, miR-146a and miR-155^15^. Previous studies have also shown that these miRNAs are involved in the regulation of T and B cell proliferation^16^,^17^. For example, miR-155 has been reported to target two important transcriptional regulators, PU.1, involved in leukemogenesis^18^, and C/EBPβ, critical for macrophage functioning^19^. Furthermore, the down-regulation of let-7 miRNA family members increases expression of cytokines IL-6 and IL-10 during *Salmonella* infection^15^. The role of miRNAs in response to *Salmonella* infection has also been investigated in pigs, and miR-29a-mediated caveolin-2 regulation has been found to control the proliferation of intestinal epithelial cells and *Salmonella* uptake^20^. Another pig study supported miR-155-mediated regulation of PU.1 and C/EBPβ in *Salmonella*-infected pigs^21^. Efforts to identify mammalian miRNAs regulated by *Salmonella* infection are now clearly under way. However, the molecular mechanisms underlying such regulation and their relevance to pathogenesis remain poorly understood especially in pigs.

Whole blood, being an easily accessible biofluid that carries cells of the immune system, is a logical sample for studying immune response-related miRNAs during pathogenic infections. Further, increasing evidence suggests that extracellular miRNAs circulate in the bloodstream and that such circulating miRNAs are remarkably stable^22^. Therefore, assessing changes in expression of miRNAs and their targets in blood on a genome-wide scale should provide a more comprehensive view of the immune response to bacterial infection. Previous studies on immunological responses and gene expression changes upon *Salmonella* challenge in pigs have identified distinct responses, with some pigs recovering faster and shedding lower levels (low shedders, LS) of *Salmonella* in faeces than others (persistent shedders, PS)^23^. Clinical differences observed as early as a day or two after inoculation were predictive of a significant difference in *Salmonella* shedding over time, with the peak of both clinical symptoms and shedding occurring at day 2 post inoculation^21^,^24^. Following a similar choice of days (days 0 and 2 post inoculation) and animals (LS and PS), this study aimed to investigate how miRNAs influence gene expression during *Salmonella* infection in pigs using genome-wide whole blood microRNAome and transcriptome analyses. The miRNAs that are identified in blood as being associated with infection or shedding status in this study may be particularly suitable as biomarkers for host responses to *Salmonella* in swine.

## Materials and Methods

### Ethics Statement

The animal care and use protocol for this study was reviewed and approved by the USDA-ARS, National Animal Disease Center, Animal Care and Use Committee (approval IDs: 3586 and 3898). All experimental procedures were in compliance with the recommended principles described in the Guide for the Care and Use of Laboratory Animals by the National Research Council of the National Academies. The blood samples used in this study were from a published challenge study conducted at the USDA National Animal Disease Center^21^.

### Sample collection and library preparation

Samples used in this study were selected from *Salmonella*-negative piglets (crossbred or Yorkshire sows bred to boars from different breeds) belonging to two populations of 40 and 77 individuals and treated as described in earlier studies^23^,^25^. In brief, the pigs were raised in climate-controlled, fully enclosed isolation facilities at the USDA-ARS-National Animal Disease Center (NADC) in Ames (IA, USA) under identical management conditions. At seven weeks of age, these *Salmonella*-negative pigs received intranasal inoculation of 10^9^ colony-forming units (cfu) of nalidixic acid-resistant *Salmonella enterica* serovar Typhimurium, ST χ4232. Approximately 2.3 ml of whole blood was collected from the jugular vein into PAXgene Blood RNA tubes (processed according to manufacturer’s instructions) from each individual just prior to *Salmonella* inoculation and at 2, 7, 14, and 20 days post inoculation (dpi). On the same days, faecal samples were also collected and the amount of *Salmonella* bacteria shed in faeces was quantified by direct counting using bacteriological methods^23^. *Salmonella* shedding status was determined based on the total amount of *Salmonella* shed in faeces calculated using the cumulative area under the plotted log curve (AULC) of the logarithmically normalised faecal counts obtained between 0 and 20 dpi for each individual as described in earlier publications^21^,^23^_ENREF_23. Based on the AULC, 16 pigs were selected for analysis of their whole blood miRNA and mRNA transcriptome, eight of which were identified as low shedders (LS) and eight as persistent shedders (PS)^21^. Peripheral whole blood (approximately 2.5 mL) was collected into PAXgene Blood RNA tubes (BD, Cat. No. 762165) during a prior challenge study conducted at the USDA National Animal Disease Center^21^. Stabilized blood from each animal at 0 and 2 dpi was processed according to the manufacturer’s instructions. Total RNA was extracted from 4.5–9.0 ml of solution from the PAXgene Blood RNA tubes using the PreAnalytiX kit (Qiagen, Cat. No. 763134). The quality and quantity of the RNA were determined using the Agilent 2100 Bioanalyzer (Agilent Technologies, Santa Clara, CA) and Qubit 2.0 Fluorometer (Invitrogen, Carlsbad, CA). All procedures involving animals were approved by the USDA-ARS-NADC Animal Care and Use Committee (approval ID: ACUP #3586).

### Illumina sequencing of miRNA and mRNAs

Total RNA (1.5 μg for each sample) was used to construct miRNA and mRNA libraries using the TruSeq Small RNA and mRNA Sample Preparation Kit (Illumina, San Diego, CA) according to the manufacturer’s instructions. Library quality for miRNA and mRNA libraries was determined using the High Sensitivity DNA Chip and an Agilent 2100 Bioanalyzer (Agilent Technologies). qRT-PCR was then performed for library quantification using the StepOne^TM^ Real-Time PCR System (Applied Biosystems, Carlsbad, CA) with the KAPA SYBR® Fast ABI Prism qPCR kit (KapaBiosystems, Woburn, MA).

The individual libraries were adjusted to 2 nM and pooled before denaturation and dilution according to Illumina’s instructions. The diluted libraries (8–10 pM) were loaded on a cBot (Illumina) for cluster generation using the TruSeq™ SR Cluster Kit v3 (Illumina). Sequencing was performed on the HiScan SQ system (Illumina) using the TruSeq™ SBS Kit v3 (50 cycles, Illumina). Real-time analysis and base calling was performed using the HiSeq Control Software version 1.4.8 (Illumina).

### Identification of miRNAs and mRNAs

miRNA analyses were performed using custom Perl scripts and miRDeep2^26^. After trimming the 3′ adaptor sequence, all sequences ranging in length from 18–26 nt were recorded in a non-redundant file along with copy number. To identify known miRNAs, the miRNA tags were aligned against miRNA precursor sequences reported in the miRNA database ‘miRBase’ (release 19) using the ‘quantifier.pl’ script within miRDeep2^26^,^27^. For novel miRNA prediction, the miRDeep2 score cutoff was set to 5 (true positive rate >90% and signal-to-noise ratio >10 at cutoff 5). Each sample was processed separately and the results for all samples were combined by genomic location. mRNA-seq reads were aligned to the pig genome assembly version Sus 10.2^28^ using Tophat 1.4.0 with default parameters^29^. The number of reads mapped to each gene was determined using htseq-count^30^.

The miRNA-seq and RNA-seq data are available in the ArrayExpress database (www.ebi.ac.uk/arrayexpress) under accession numbers E-MTAB-2286 and E-MTAB-2234, respectively.

### DE analysis of miRNAs and mRNAs

DE analysis of miRNA and mRNA sequence data was performed with the Bioconductor package edgeR, which is designed for use with digital gene expression data^31^. Read counts were imported to edgeR, log2 transformed, and normalized based on the negative binomial distribution to obtain normalized expression levels as read counts per million mapped reads (cpm). We required cpm >= 2 in at least 7 samples for identification of expressed miRNAs and mRNAs in blood. DE was evaluated by fitting a negative binomial generalized linear model and then adjusting the P-value for multiple testing using the Benjamini-Hochberg correction with a false discovery rate of 0.1 for miRNA and mRNA.

### Prediction of miRNA targets and construction of miRNA-mRNA regulatory networks

Our strategy for identifying miRNA-mRNA regulatory relationships was based on two criteria: computational targets prediction and negative regulation relationship. Because pig data was not available in TargetScan, miRanda^32^ was used for computational target prediction. This software predicts target genes based on sequence complementarity and the free energy of the RNA duplex. We required an alignment score >145 and energy <−10 kcal/mol, as suggested by Zhang^33^. An R script (miRCausality.R) was used for identification of causal miRNA-mRNA regulatory relationships^34^. The method learns a causal structure from expression data, and applies *do-calculus*^35^ to infer regulatory effects (range from −1 to 1).We calculated pairwise causal effects between each DE miRNA and mRNA based on their expression across all samples. A regulatory effect less than −0.3 was considered to represent a negative regulatory relationship.

### miRNA target validation

The 3′UTR of *SLC11A1* (previously known as *NRAMP1*) and PIGE-108A11.3 (leukocyte immunoglobulin-like receptor-like; henceforth referred to as *LILR-like*) containing miR-214 binding site and the 3′UTR of *VAV2* containing miR-331-3p binding site were amplified from pig genomic DNA by PCR using the following primer pairs: GCTAGCGCGGCCGCATCCAAGCAGGCAGACAGAAA (forward) and GTCGACCCCCTTCTTCTGGAGGTGTT (reverse) for *SLC11A1*; GCTAGCGCGGCCGCGTTCAGAGTGGCAGAGCCTT (forward) and GTCGACTCTGTGTTCTGGGATGGATGG for *LILR-like* (reverse); and GCTAGCGCGGCCGCCAGAGCTGGAGCGACTCTTC (forward) and GTCGACGAGCCACCAGGGAACTCCA for *VAV2* (reverse). All three PCR products were cloned into pmirGLO Dual-Luciferase miRNA Target Expression Vector (Promega, Madison, USA) using the Xhol and Sall restriction sites.

The pig kidney cell line (PK-15, ATCC® CCL-33) was cultured in ATCC-formulated Eagle’s Minimum Essential Medium (Catalog No. 30-2003) supplemented with 10% fetal bovine serum (Gibco, Invitrogen, Carlsbad, CA, USA), at 37 °C and with 5% CO2. Exactly 30 pmol miRNA mimics (miR-214/miR331-3p/negative control mimic) were separately transfected into the cell line with with 200 ng luciferase reporter containing target genes’ 3′UTR using 2 μl Lipofectamine 2000 reagent (Invitrogen, Carlsbad, CA, USA) and 24-well plates. The negative control used in this study was mirVana™ miRNA Mimic Negative Control #1 (Ambion, Carlsbad, CA, USA), a random sequence miRNA mimic molecule that has been extensively tested in human cell lines and tissues and validated to not produce identifiable effects on known miRNA function. The cell line was incubated for 48 hours after transfection, and then the Dual-Glo luciferase assay system (Promega, Madison, USA) and SpectraMax M3 microplate reader were used to measure the quantity of firefly and Renilla luciferase. The firefly luciferase quantity was first normalized to their matching Renilla luciferase quantity. Those ratios were then normalized to empty vector controls.

## Results

### miRNA and mRNA profiles in the whole blood of pigs

To determine the miRNA and mRNA expression patterns in response to *Salmonella* challenge in pigs, the miRNA and mRNA transcriptome were characterized by high-throughput sequencing using whole blood samples collected at 0 dpi and 2 dpi from eight LS and eight PS pigs, which were so classified in previous studies (See Methods and Supplementary Table S1). The LS had an average AULC of 68 while the PS had an average AULC of 159 based on accumulated shedding counts up to 20 dpi. PS pigs maintained higher levels of shedding longer, whereas shedding in LS pigs quickly decreased to low levels.

We obtained an average of 8 million reads per miRNA sample, approximately 89% of which could be mapped to the pig reference genome assembly version Sus 10.2^28^. One animal from the LS group was excluded from analysis due to low sequencing coverage. Based on miRDeep2 (for miRNA identification) and edgeR (for miRNA expression) analysis, we identified 308 miRNAs expressed (cpm > =2 in at least 7 samples) in blood, including 181 known miRNAs and 127 novel miRNAs (Supplementary Table S2).

In parallel, we obtained 23–52 million single-end 50-bp reads from each mRNA sample, approximately 90% of which could be aligned to the pig genome. A total of 10936 protein-coding genes were identified as expressed in the pig whole blood (cpm > =2 in at least 7 samples).

### Differential expression of miRNAs in response to *Salmonella* infection

To visualize the overall pattern of miRNA expression among samples, we conducted multidimensional scaling (MDS) analysis. The MDS plot displays the position of each sample in two-dimensional Euclidean space, with the distance between samples reflecting their approximate degree of correlation. MDS analysis using all expressed miRNAs revealed a clear separation between the 0 dpi and 2 dpi samples but no separation was observed between the LS and PS samples on both days (Supplementary Fig. S1). Whole blood contains different cell populations and changes in miRNA expression could reflect changes in specific cell populations rather than changes in cell-specific expression levels^21^. As performed in a previous study on gene expression in porcine whole blood^21^, we analyzed the correlation between blood cell type counts (lymphocyte, monocyte, neutrophil, eosinophil, and basophil counts) and the expression levels of miRNAs. No significant correlations were observed (at false discovery rate (FDR) below 0.01 computed from *p*-values using the Benjamini-Hochberg (BH) procedure), supporting the notion that cell count changes do not account for the differences in expression of the miRNAs at least in this study.

**Figure 1:**
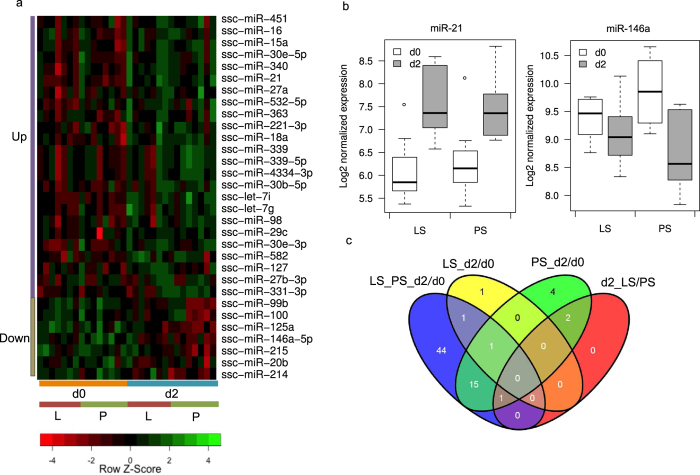
Differentially expressed (DE) miRNAs in response to *Salmonella* infection. (**a**) Heat map of DE known miRNAs (FDR < 0.01) (**b**) Bar plot showing expression change of the top five DE miRNAs (**c**) Venn diagram displaying the overlap among different groups of DE miRNAs.

A linear model-based statistical analysis of 15 samples (7 LS + 8PS) before and after *Salmonella* infection identified 62 differentially expressed (DE) miRNAs (38 up- and 24 down-regulated) at FDR < 0.1 (Table 1). A representative heat map of a subset (FDR < 0.01) of known miRNAs that were DE upon infection is shown in Fig. 1A. Several miRNAs previously reported to be involved in immune response such as miR-21, miR-125a, miR-99b, miR-146a and let-7 families were identified. For example, ssc-miR-21 was significantly up-regulated (log2fold change (logFC) = 1.31, FDR < 1E-6, Fig. 1B), and ssc-miR-146a was significantly down-regulated (logFC = −0.91, FDR < 1E-5, Fig. 1B) at 2 dpi. We also identified several new candidate miRNAs associated with *Salmonella* infection such as ssc-miR-30e-3p (up-regulated, FDR = 0.003) and ssc-miR-214 (down-regulated, FDR = 0.0002). In addition to the known miRNAs, a number of novel (not annotated in miRBase 19) miRNAs were also significantly DE. The changes in miRNA expression after challenge were significantly less dramatic in LS compared to PS samples. While 23 miRNAs were DE in PS samples (Table 1), only three were DE in LS (miR-21, miR-340, miR-148a). A Venn diagram displaying the overlap among miRNAs found DE in the various comparisons performed is provided in Fig. 1C. For the comparisons between LS and PS, no miRNAs were DE at 0 dpi and only three were DE at 2 dpi. However, these three miRNAs were not unique to the comparison between LS and PS at 2 dpi but were also found in the 2 dpi versus 0 dpi comparisons in either LS or PS. Hence we do not find any biomarkers specific to shedding status prediction from miRNA expression at either 0 dpi or 2 dpi. Complete lists of DE miRNAs (both known and novel) for various comparisons are provided in Supplementary Table S3.

### Differential expression of mRNAs in response to *Salmonella* infection

**Figure 2:**
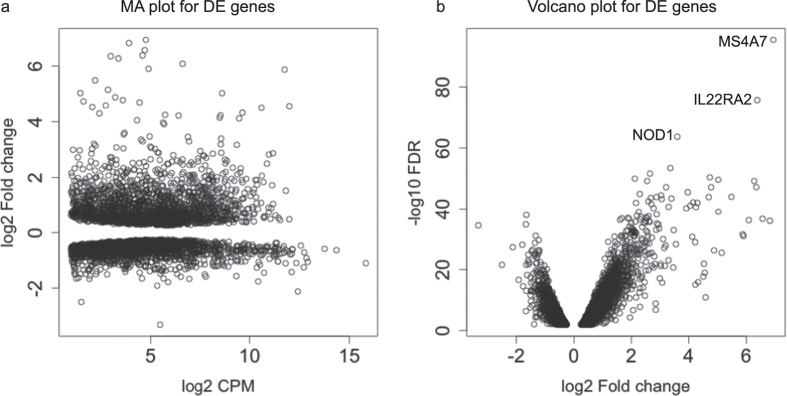
Differentially expressed (DE) mRNAs in response to *Salmonella* infection. MA plot (**a**) and volcano plot (**b**) showing DE mRNAs after *Salmonella* infection. The names of top three most significant DE genes are shown.

We analyzed the correlation between blood cell type count (lymphocyte, monocyte, neutrophil, eosinophil, and basophil counts) and the expression levels of mRNAs, and observed that the expression levels of 75 mRNAs were positively correlated (BH corrected p < 0.01) with one or more blood cell type counts and these mRNAs were excluded from further analyses. The mRNA data also revealed dramatic mRNA expression changes after challenge but few DE mRNAs between LS and PS samples. When analyzing LS and PS samples together, a total of 4443 DE genes were identified between 0 dpi and 2 dpi (Table 1, Fig. 2). Compared to the PS samples (4136 DE genes), the changes in mRNA expression of LS samples (1584 DE genes) were less dramatic, which is also consistent with the results from the miRNA analysis (Table 1) and with a previous microarray-based study^21^. However, the degree of mRNA expression change is considerably higher compared to that of miRNA. The top 10 most significant DE mRNAs show an average logFC of 4.3, whereas the top 10 miRNAs only have logFC of 1.16. Several immune-related genes were found significantly DE after challenge. For example, the expression of *IL22RA2* and *NOD1* was significantly up-regulated following *Salmonella* challenge (logFC 6.38 and 3.60, respectively). Complete lists of differentially expressed mRNAs are provided in Supplementary Table S4.

Though about 50% of mammalian miRNAs are located within protein-coding genes, the correlations between intragenic miRNA and host gene expression profiles have generally been poor mainly due to the fact that over a third of the intronic miRNAs have their own promoters that regulate their expression independent of the promoters of host genes^36^,^37^,^38^. Based on the genomic positions of mature miRNAs and mRNAs that we identified here as expressed, we determined intragenic miRNAs and tested the Spearman correlations of expression among those miRNAs and their host genes at 0 and 2 dpi (Supplementary Table S5). Of 42 matched miRNA-mRNA expression profiles, only seven and nine showed low to moderate correlations (0.3< correlation <0.65) at 0 and 2 dpi respectively. Therefore, the use of expression profiles of host genes as a proxy for the expression of the corresponding intragenic miRNAs should be treated with caution, as has been suggested earlier^38^.

### Identification of miRNA-mRNA regulatory interactions associated with *Salmonella* infection

Targets of miRNAs were identified based on sequence complementary and free energy of the predicted RNA duplex. Next, we analyzed the negative regulation relationship between miRNAs and predicted target mRNAs. Several previous studies predicted miRNA targets based on the anti-correlations between miRNA and mRNA expression levels^39^,^40^,^41^. However, such correlations are not indicative of causality; instead, they can be the result of the mRNA regulating the miRNA, or a third molecule regulating both the miRNA and the mRNA^34^. A recently developed R package called miRCausality^34^ that aims to capture causal effects was used to infer the miRNA-mRNA regulatory relationships from the expression data. The regulatory effect (from −1 to 1) of a miRNA on an mRNA reflects its approximate degree of regulation (knockdown or over-expression)^34^. This analysis when applied to all DE miRNAs and DE mRNAs identified 1608 putative miRNA-mRNA regulatory pairs (regulatory effects <−0.3) containing 69 DE miRNAs and 936 DE mRNAs. These miRNAs and mRNAs are listed in Supplementary Table S6 along with their associated gene ontology terms, obtained using the Bioconductor package ‘biomaRt’^42^. This approach identified several known miRNA-mRNA interactions, for example, *SPI1* and *SOCS1* as the targets of miR-155 (regulatory effects −0.32 and −0.30), and *BAK1* as the target of miR-125a (regulatory effect −0.36). In order to assess the biological effects of the 936 targets of 69 DE miRNAs, we looked for enriched gene ontology biological processes using Bioconductor package ‘GOstats’^43^. These targets were found to be enriched in signaling, cell communication and immune-related processes. The most significant enriched terms are shown in Table 2.

### The potential effects of miRNA-mRNA interactions on immunological functions

**Figure 3:**
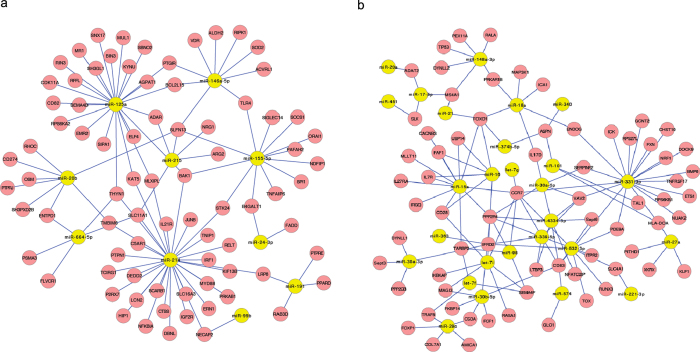
Immune-related miRNA-mRNA interactions associated with infection. (**a**) miRNA-mRNA network among down-regulated miRNAs and up-regulated mRNAs (**b**) miRNA-mRNA network among up-regulated miRNAs and down-regulated mRNAs.

To further illustrate the immunological significance of the miRNA-mRNA interactions, we downloaded a list of immune-related genes (6004 human genes) from the IMMPORT database (https://immport.niaid.nih.gov) and determined that the human orthologues of 253 of the 936 porcine miRNA targets identified in this study were annotated as immune-related in the IMMPORT database. Based on the putative miRNA-mRNA regulatory pairs identified from the miRCausality analysis, it was found that the 253 immune-related genes can be targeted by 60 of the 69 DE miRNAs (Supplementary Table S7). The immune-related miRNA-mRNA interaction networks are shown in Fig. 3A,B. We further examined expression data for genes that are representative of canonical immune pathways such as T cell-mediated immune responses, inflammation, and apoptosis. The mRNA levels for innate/inflammatory marker genes such as *SPI1* were strongly up-regulated following *Salmonella* infection (data not shown). *SPI1* is a known target of miR-155^13^ and thus the down-regulation of miR-155 is consistent with the increased expression of *SPI1* after *Salmonella* challenge. *BAK1* and *BCL2* (apoptosis pathway genes) are known targets of miR-125a^44^. In our study, the expression of *BAK1* and *BCL2* were significantly increased after challenge, consistent with the down-regulation of miR-125a.

In addition to the known miRNA-mRNA interactions, several novel interactions involving immune-related pathways were identified. Table 3 summarizes the predicted interactions and the immune-related pathways that are potentially affected. Some mRNAs are highly connected and regulated by multiple miRNAs. *CCR7*, for example, is involved in cytokine-cytokine receptor interaction and was identified as a potential target of let-7g, miR-15a, miR-98 and miR-331. *VAV2* is involved in chemokine signaling and was predicted to be regulated by miR-30a, miR-331 and miR-339.

Two *Salmonella*-regulated miRNAs of particular interest identified through this study are miR-214 and miR-331-3p. The expression levels of miR-214 (log2cpm = 2.25 across all samples) and miR-331-3p (log2cpm = 5.62 across all samples) are relatively high. Further, both are highly connected (>15 immune-related target genes) within the miRNA-mRNA network, have not been previously reported to be associated with *Salmonella* infection, and are predicted to regulate several immune-related genes. For these reasons we further studied the expression of these miRNAs and their targets.

### Validations of miRNA-mRNA interactions using miR-214 -*SLC11A1*, miR-214 -*LILR-like* and miR-331-3p -*VAV2* mimics

**Figure 4:**
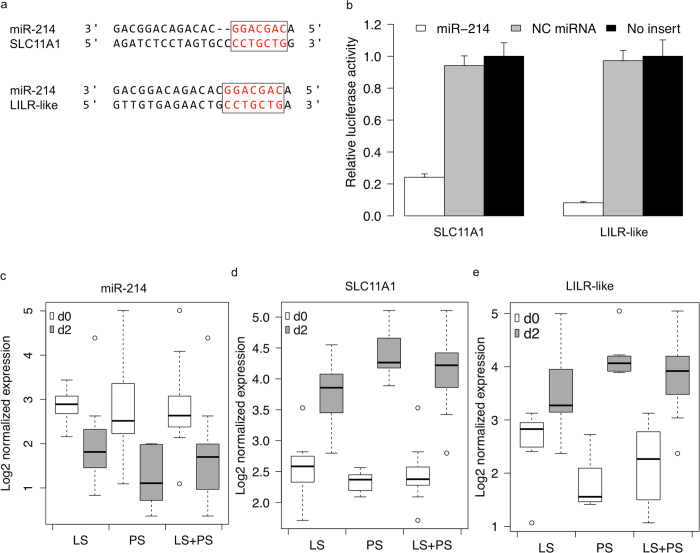
Regulation of *SLC11A1* and *LILR*-like by miR-214. (**a**) *SLC11A1* and *LILR-like* contain miR-214 seed (**b**) Luciferase activity in pig kidney cells transfected with miRNA mimics and plasmids carrying the 3’ UTR of SLC11A1 or LILR-like. NC miRNA = negative control (scrambled) miRNA. (**c**) Expression change of miR-214 after infection (**d**) Expression change of SLC11A1 after infection (**e**) Expression change of LILR-like after infection.

*SLC11A1* and *LILR-like* have critical roles in immune responses to intracellular bacterial infection^45^,^46^. However, whether miRNAs can directly target and regulate expression of these genes remains unknown. Our bioinformatics analysis revealed that the 3′ UTRs of pig *SLC11A1* (ENSSSCG00000025058) and *LILR-like* (ENSSSCG00000003282) each contain one binding site complementary to the seed sequence of miR-214 (Fig. 4A). Further, miR-214 and these two genes showed a negative regulatory effect of -0.34. The expression of miR-214 was down-regulated in LS (logFC 0.77), PS (logFC 1.54) and LS + PS (logFC 1.18) after *Salmonella* challenge (Fig. 4C). In contrast, *SLC11A1* showed up-regulation in LS (logFC 1.18), PS (logFC 2.06) and LS + PS (logFC 1.65) after challenge (Fig. 4D). *LILR-like* also showed up-regulation in LS (logFC 0.99), PS (logFC 2.37) and LS + PS (logFC 1.72) after challenge (Fig. 4E).

**Figure 5:**
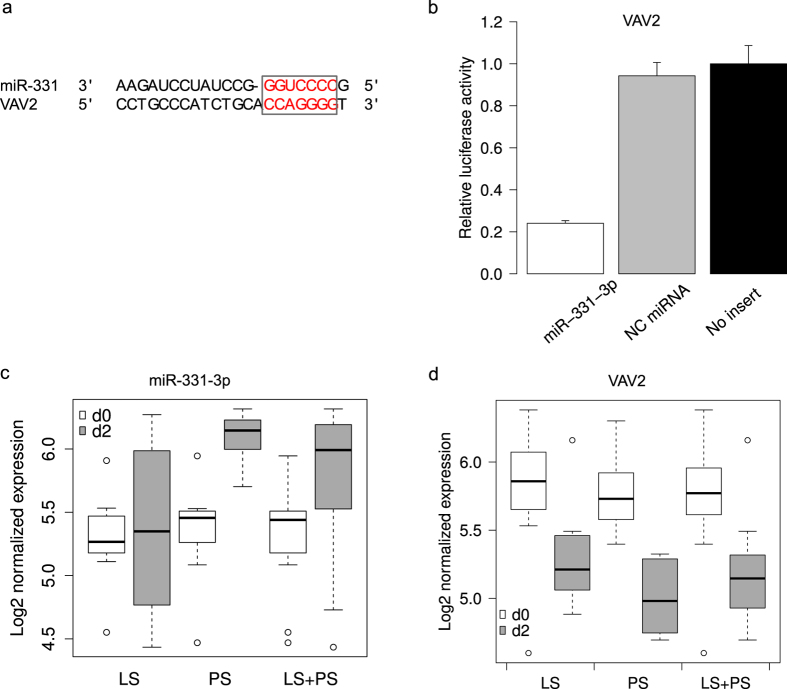
Regulation of *VAV2* by miR-331-3p. (**a**) VAV2 contains miR-331-3p seed (**b**) Luciferase activity in pig kidney cells transfected with miRNA mimics and plasmids carrying the 3’ UTR of VAV2. NC miRNA = negative control (scrambled) miRNA (**c**) Expression change of miR-331-3p after infection (**d**) Expression change of VAV2 after infection.

*VAV2* has been shown to play a direct role in the Cav1-mediated prevention of bacterial uptake^47^. However, whether miRNAs can directly target and regulate its expression remains unknown. The 3′ UTR of the pig *VAV2* (ENSSSCG00000005743) RNA was predicted to contain one binding site complementary to the seed sequence of miR-331-3p (Fig. 5A). Further, the expression of miR-331-3p was up-regulated in LS (logFC 0.08), PS (logFC 0.74) and LS + PS (logFC 0.43) after *Salmonella* challenge (Fig. 5C). *VAV2* showed down-regulation in LS (logFC 0.43), PS (logFC 0.76) and LS + PS (logFC 0.60) after challenge (Fig. 5D).

To validate the predicted interactions stated above, we used the luciferase reporter gene system. We cloned the 3′ UTRs of *SLC11A1*, *LILR-like* and *VAV2* into luciferase reporter plasmids to test miR-214 and miR-331-3p functions *in vitro*. Transfection with an miR-214 mimic resulted in significant (p < 0.01, t test) reduction in relative luciferase activity for both *SLC11A1* and *LILR* plasmids (Fig. 4B), compared with negative control miRNA (random miRNA sequence) and no-insert control. Similarly, transfections with mimics resulted in significant (p < 0.01, t test) reduction in relative luciferase activity for *VAV2* (Fig. 5B) compared with negative control miRNA (random miRNA sequence) and no-insert control. These results may indicate that similar responses are happening in the host during *Salmonella* infection, that is, the down-regulation of miR-214 may allow for increased expression of *SLC11A1* and *LILR-like* during *Salmonella* infection, and up-regulation of miR-331-3p expression may inhibit expression of *VAV2*.

## Discussion

miRNAs are important regulators of innate and adaptive immunity^13^. However, their specific roles in regulating the response to *Salmonella* infection are still poorly understood. We present a systematic study of miRNA and mRNA profiles from whole blood of pigs upon *Salmonella* challenge. This study identified differential expression of several miRNAs previously linked to immune response including miR-21, miR-146a and miR-125a^13^, and reported several miRNAs not previously linked to immune response to *Salmonella* infection, such as miR-214, miR-30e-3p and miR-331. These miRNAs are valuable candidate biomarkers and potential regulators of host immune responses to *Salmonella* infection. However, we did not find any candidate biomarkers specific to *Salmonella* shedding status prediction from miRNA expression at either 0 dpi or 2 dpi.

Through the integration of miRNA and mRNA expression data and miRNA-RNA target prediction analysis, a large number of putative miRNA-mRNA interactions were identified. Notably, our analysis has predicted immune-related pathway targets for many miRNAs. For example, miR-24, miR-146a, miR-155 and miR-214 were predicted to be involved in regulation of the Toll-like receptor (TLR) signaling pathway (Table 3). Further, miR-18a, miR-24, miR-146a, miR-148a and miR-214 were predicted to target the apoptosis pathway (Table 3).

Since hub nodes have been found to play important roles in many networks^48^, we also looked for the presence of hub miRNAs. Several miRNA hub nodes were identified including miR-146a, miR-155, miR-214 and miR-331-3p (Fig. 3). It has been shown that miR-146a and miR-155 are involved in the TLR signaling pathway and play important roles in innate immune response^13^. A previous study showed that mice deficient in both Toll-like receptor 2 (*TLR2*) and *TLR4* were highly susceptible to *Salmonella*^49^. In contrast, miR-214 and miR-331-3p have not been previously linked to *Salmonella* infection. As miR-214 and miR-331-3p were predicted to target several important immune-related genes, our study focused on these two miRNAs and three of their targets, *SLC11A1*, *LILR-like*, and *VAV2* which are known to play critical roles in immune responses to intracellular bacterial infection^45^,^46^,^50^. Our further discussion is focused on these two miRNAs and three of their target genes.

The protein encoded by *SLC11A1* regulates intracellular pathogen proliferation and macrophage inflammatory responses by controlling intracellular iron homoeostasis^51^. It has been proposed to have a role in iron recycling, removing iron and iron-containing compounds from the macrophage after phagocytosis of dead red blood cells. It controls the innate resistance to infection in mice by a group of intracellular parasites including *Salmonella*, *Leishmania*, and *Mycobacterium*^45^. A previous study showed that the mRNA level of *SLC11A1* increases following *Mycobacterium* infection in mice^52^. It has been shown that transcriptional factors Sp1 and C/EBP can regulate *SLC11A1* expression^53^. However, to our knowledge, there is no report demonstrating a direct role for miRNA in control of the expression of *SLC11A1* at the post-transcriptional stage. Here, we identify *SLC11A1* as a new immune-related target of miRNA. Members of the *LILR* family are innate immune receptors for self-proteins. *LILR*, expressed in monocytes, can regulate TLR activity and the antigen presenting cell phenotype^54^. TLRs act as innate immune receptors for microbes and trigger an immune response to non-self, whereas LILRs acting as innate immune receptors for self could provide an inhibitory balancing force^55^. As the immune system needs to constantly strike a balance between activation and inhibition to avoid detrimental inflammatory responses, TLR signaling must be tightly regulated^56^. Although relatively little is known about the function of LILR, it is becoming clear that this family of receptors, with its ability to constrain the effects of TLR signaling, could have far-reaching effects on immune response^55^. Our study revealed a negative causal effect between miR-214 (down-regulated) and *SLC11A1* and *LILR-like* (both up-regulated) *in vivo*, and that miR-214 can directly target both *SLC11A1* and *LILR-like in vitro*.

Invasion of host target cells is the first stage of infection by several pathogenic microbes. The entry process often involves the activation of small Rho family GTPase including RhoA, Cdc42 and Rac1, which act as guanine nucleotide-regulated switches to induce various responses during the infection process^57^. It has been shown that Vav2 is an activator of RhoA, Cdc42 and Rac1^58^ and that down-regulation of *VAV2* led to a significant drop in the amount of intracellular bacteria *Campylobacter jejun*^59^. Our study shows that *VAV2* is significantly down-regulated after *Salmonella* infection *in vivo* and that it can be targeted by miR-331-3p *in vitro*.

Based on our observations noted above, we propose potential roles for miR-214 and miR-331-3p in the regulation of immune responses to *Salmonella* infection. We hypothesize that decreased miR-214 expression in whole blood after *Salmonella* challenge may help boost the host immune response in two ways: one, by allowing increased *SLC11A1* expression which in turn controls *Salmonella* replication by actively removing iron from the phagosomal space and two, by allowing increased *LILR-like* expression which in turn negatively regulates TLR-mediated immune response to maintain immunological balance. Similarly, the observed increased expression of miR-331-3p could favor the host immune response against *Salmonella* through the down-regulation of *VAV2* to help block *Salmonella* uptake by suppressing of GTPases activities. Future research examining the precise temporal and spatial expression patterns of these genes and the effects of changes in their expression on *Salmonella* infection is warranted.

To conclude, the deep sequencing of the transcriptome and microRNAome from the whole blood in pigs before and after *Salmonella* challenge revealed that *Salmonella* infection in pigs caused significant changes in miRNA and mRNA expression. We identified 253 immune-related DE genes involved in a variety of processes including T cell-mediated immune responses, inflammation, and apoptosis and 60 DE miRNAs that target them. Further, we identified two miRNAs, miR-214 and miR-331, occupying hub positions in the miRNA-mRNA regulatory network that could potentially target *SLC11A1* and *LILR-like*, and *VAV2*, respectively. These two miRNAs are candidate biomarkers associated with host response mechanisms during *Salmonella* infection. These sequence data and expression analyses should contribute to a better understanding of the miRNA-mediated regulation of host genes during *Salmonella* pathogenesis in swine, and could lead to new approaches for diagnosis and prevention of the transmission of this human foodborne pathogen from farm animals to humans.

## Additional Information

**How to cite this article**: Bao, H. *et al.* Genome-wide whole blood microRNAome and transcriptome analyses reveal miRNA-mRNA regulated host response to foodborne pathogen *Salmonella* infection in swine. *Sci. Rep.* **5**, 12620; doi: 10.1038/srep12620 (2015).

## Supplementary Material

Supplementary Information

Supplementary tables

## Figures and Tables

**Table 1:** Number of differentially expressed miRNAs and mRNAs.

Comparison	DE miRNAs (FDR < 0.1)			DE mRNAs (FDR < 0.1)		
	TOTAL	UP	DOWN	TOTAL	UP	DOWN
2 dpi vs 0 dpi (LS_PS_d2/d0)	62	38	24	4443	2117	2326
2 dpi vs 0 dpi in LS (LS_d2/d0)	3	3	0	1584	1072	512
2 dpi vs 0 dpi in PS (PS_d2/d0)	23	7	16	4136	1959	2177
LS vs PS at 0 dpi (d0_LS/PS)	0	0	0	3	2	1
LS vs PS at 2 dpi (d2_LS/PS)	3	0	3	20	19	1

**Table 2:** Gene ontology enrichment analysis of miRNA target genes.

Biological Process	No. genes	Bonferroni P-value
Regulation of signal transduction	143	0.000177
Response to external stimulus	99	0.000405
Regulation of cell communication	153	0.000559
Regulation of signaling	152	0.000804
Cell communication	287	0.003609
Intracellular signal transduction	150	0.007701

**Table 3:** miRNAs and predicted targets involved in immune-related pathways.

Pathway	Genes	miRNAs
Toll-like receptor signaling pathway	*RIPK1,NFKBIA,TLR4,CXCL10,MAPK1,MYD88, RELA,PIK3R5,MAPK3,TLR6,RAC1,FADD*	miR-24, miR-146a, miR-155, miR-214
Chemokine signaling pathway	*NFKBIA,VAV2,LYN,CXCL10,MAPK1,GNAI2,ADRBK1, RELA,STAT5B,CCR7,PIK3R5,MAPK3,XCR1,GNG2,RAC1*	let-7g, miR-15a, miR-98, miR-214, miR-331, miR-339
Apoptosis	*CSF2RB,RIPK1,NFKBIA,ENDOG,PRKAR1B,MYD88, RELA,CFLAR,TP53,PIK3R5,FADD*	miR-18a, miR-24, miR-146a, miR-148a, miR-214
TNF signaling pathway	*RIPK1,NFKBIA,CXCL10,MAPK1,RELA,JUNB,TRAF5, CFLAR,PIK3R5,MAPK3,TNFRSF1B,FADD*	let-7f, miR-24, miR-146a, miR-214
B cell receptor signaling pathway	*NFKBIE,NFKBIA,VAV2,LYN,MAPK1, RELA,INPPL1,PIK3R5,MAPK3,RAC1*	miR-30a, miR-214, miR-331, miR-339
Cytokine-cytokine receptor interaction	*CSF2RB,LTBR,IL21R,TNFRSF17,KIT,CXCL10,OSM,IL2RG, RELT,IL7R,CCR7,IL15RA,TNFRSF1B,XCR1*	let-7g, miR-15a, miR-16, miR-98, miR-214, miR-331
