# Supplementary material for: Genome-wide whole blood microRNAome and transcriptome analyses reveal miRNA-mRNA regulated host response to foodborne pathogen Salmonella infection in swine
Source: Sci Rep. 2015 Jul 31;5:12620. doi: 10.1038/srep12620 (PMC4521145; doi:10.1038/srep12620)
Supplement: Supplementary Information [file srep12620-s1.docx]

**Genome-wide whole blood microRNAome and transcriptome analyses revealed miRNA-mRNA regulated host response to foodborne pathogen *Salmonella* infection in swine**

Hua Bao^1§^, Arun Kommadath^1§^, Guanxiang Liang^1^, Xu Sun^1^, Adriano S. Arantes^1^, Christopher K. Tuggle^2^, Shawn M.D. Bearson^3^, Graham S. Plastow^1^, Paul Stothard^1*^, Le Luo Guan^1*^

^1^Department of Agricultural, Food and Nutritional Science, University of Alberta, Edmonton, Alberta, Canada T6G2P5

^2^Departmentof Animal Science, Iowa State University, Ames, IA, United States, 50011

^3^Food Safety & Enteric Pathogens Research Unit, USDA/ARS/National Animal Disease Center, Ames, Iowa, USA, 50010

^§^These authors contributed equally to the current work

^*^Corresponding authors

**Supplementary Figures**

**Supplementary Figure S1:** Multi-dimensional scaling plot of miRNA expression from all samples.

**Supplementary Table Legends**

**Supplementary Table S1:** Sample information.

**Supplementary Table S2:** Expression levels of known and novel miRNA in counts per million (CPM).

**Supplementary Table S3:** List of differentially expressed miRNAs for different contrasts.

**Supplementary Table S4:** List of differentially expressed mRNAs for different contrasts.

**Supplementary Table S5:** Spearman correlations between intragenic miRNA and their host genes at 0 and 2 dpi.

**Supplementary Table S6:** List of all miRNA-mRNA interactions identified here and their associated gene ontology terms.

**Supplementary Table S7:** List of immune-related miRNA-mRNA interactions identified here.
